# Suture Bridge Fixation for Posterior Cruciate Ligament Tibial Avulsion Fracture in Children

**DOI:** 10.1016/j.eats.2021.12.012

**Published:** 2022-03-19

**Authors:** Tomoyuki Kanayama, Junsuke Nakase, Kazuki Asai, Rikuto Yoshimizu, Mitsuhiro Kimura, Hiroyuki Tsuchiya

**Affiliations:** Department of Orthopaedic Surgery, Graduate School of Medical Sciences, Kanazawa University, Kanazawa, Japan

## Abstract

Posterior cruciate ligament (PCL) tibial avulsion fractures in children are extremely rare. Due to the rarity of these injuries, careful attention to the specific physical examination and imaging findings is necessary for a proper diagnosis. PCL avulsion fractures can be missed on plain radiography in skeletally immature patients. Magnetic resonance imaging should be considered if sagging or posterior drawer sign is positive after a strong hit to the anterior aspect of the lower leg. With this knowledge, clinicians can formulate treatment plans that can return patients to their original functionality while avoiding potential morbidity from misdiagnoses. We treated these patients using the suture bridge method. In children, ossification is incomplete, and they possess a lot of cartilage, so screw fixation easily destroys avulsed fragment. The suture bridge method can firmly fix the avulsed fragments, reducing the risk of damage to the bone fragment; therefore, a secondary surgery for implant removal is not needed. Arthroscopic surgery also was expected to be technically difficult in children due to the limited scope of the operation. We used open fixation because the outcome was unaffected by open surgery and arthroscopic surgery, and all patients returned to full sporting activity postoperatively.

Posterior cruciate ligament (PCL) tibial avulsion fractures are rare in the pediatric population. Ross and Chesterman^1^ first reported a case of pediatric tibial avulsion fracture of the PCL in 1986. The injury mechanism is known to result from a direct external force to the proximal anterior surface of the tibia or external forces such as hyperextension, hyperflexion, or strong external rotation. Furthermore, the relative weakness of the physis and bone in comparison with the ligament in children more commonly results in osteochondral avulsions rather than ligamentous substance tears, as seen in adults.^2^ PCL avulsion fractures can be missed on plain radiographs in skeletally immature patients. In children, ossification is incomplete and they possess a lot of cartilage, so screw fixation easily destroys the bone fragment. The suture bridge method can firmly fix the bone fragments. Furthermore, the risk of damage to the bone fragment is less, and a secondary surgery for implant removal is not needed.

## Surgical Technique (With Video Illustration)

**Fig 1:**
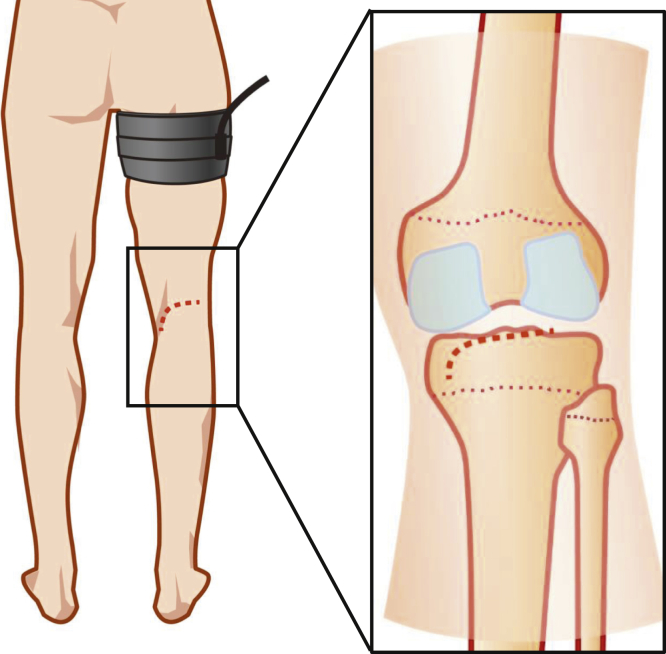
Positioning the patient. The right knee is placed in a prone position on a standard operating table. A sterile tourniquet is placed at the upper thigh of the leg to be operated. Surgery is performed using the modified Burks approach. A skin incision is made by smoothly curving the lateral limb just below the crease of the hind knee to the vertical limb along the medial head of gastrocnemius muscle (dashed line).

**Fig 2:**
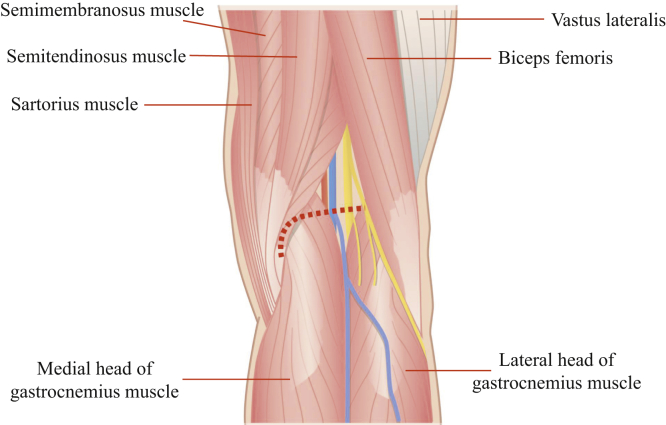
Development of the superficial layer. The right knee is placed in a prone position on a standard operating table. A blunt incision is made in the deep fascia and extended along the medial head of the gastrocnemius muscle (dashed line).

The surgical technique is shown in Video 1. The patient is placed in the prone position on a standard operating table. A sterile tourniquet is then placed on the upper thigh of the leg to be operated. Surgery is performed using the modified Burks approach. A skin incision is made by smoothly curving the lateral limb just below the crease of the hind knee to the vertical limb along the medial head of the gastrocnemius muscle (Fig 1). Afterwards, blunt incision into the deep fascia is progressed along the medial head of the gastrocnemius muscle (Fig 2). Developing a plane between the semimembranosus tendon and the medial head of the gastrocnemius muscle, the latter laterally protects the popliteal vessels into the gastrocnemius muscle.

**Fig 3:**
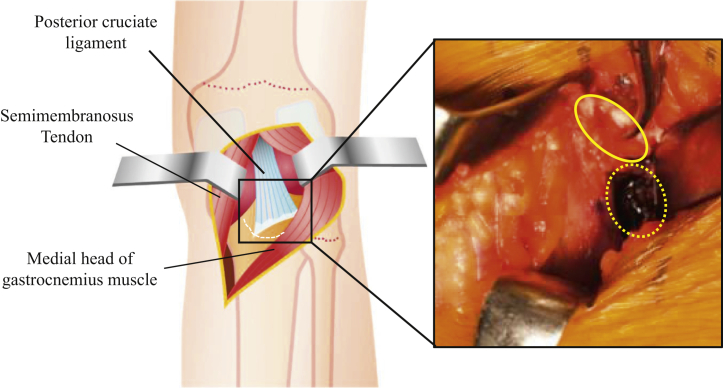
Identification of the avulsed site. The right knee is placed in a prone position on a standard operating table. Developing a plane between the semimembranosus tendon muscle and the medial head of the gastrocnemius muscle, the latter laterally protects the popliteal vessels into the gastrocnemius muscle. After incising the dorsal root of the oblique popliteal ligament and the joint capsule outside the dorsal root of the medial meniscus, the avulsed fragment (solid circle) and the footprint (dashed circle) can be identified.

**Fig 4:**
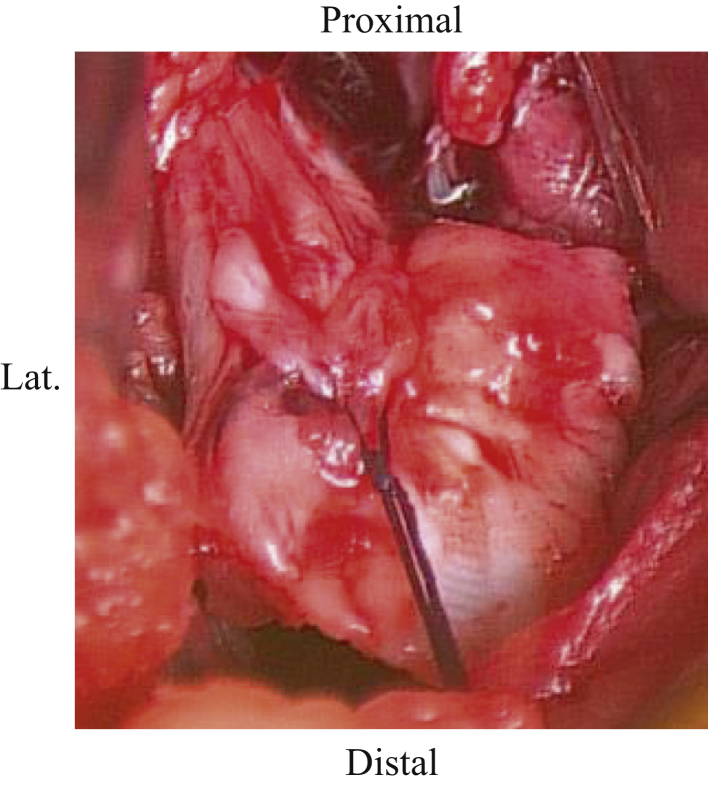
Confirming the viability of reduction. The right knee is placed in a prone position on a standard operating table. After dissecting the footprint and the avulsed fragment, the possibility of reduction is confirmed by threading the avulsed fragment and pulling it.

**Fig 5:**
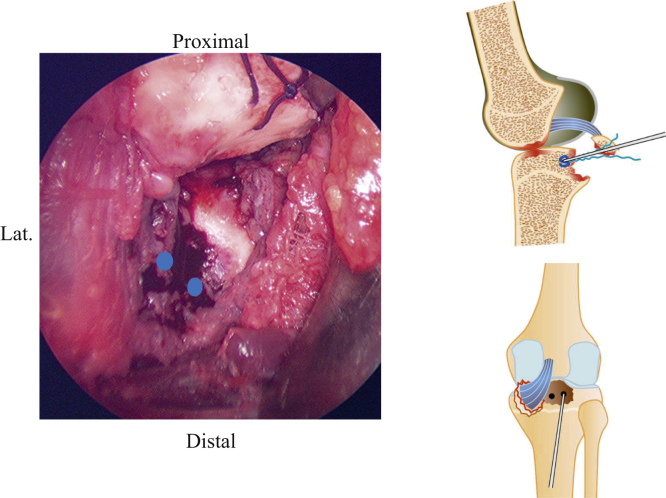
Insertion anchors. The right knee is placed in a prone position on a standard operating table. Two soft anchors (solid circles) are placed on the footprint.

**Fig 6:**
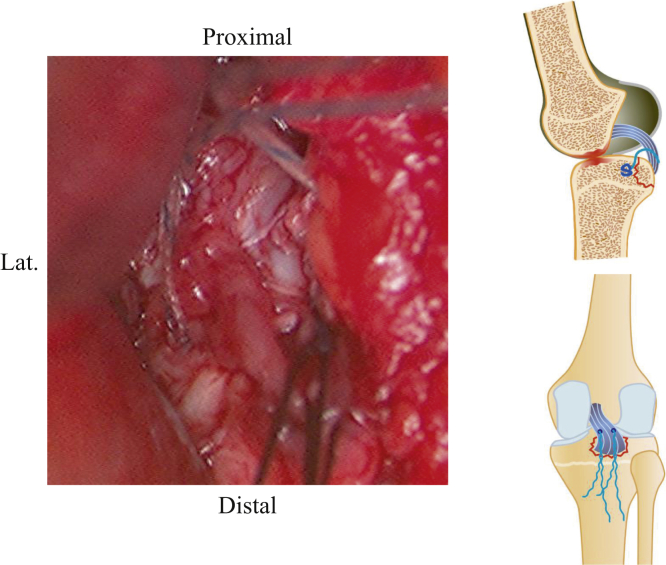
Passing the suture between the PCL and the avulsed fragment. The right knee is placed in a prone position on a standard operating table. The suture is passed between the PCL and the avulsed fragment. (PCL, posterior cruciate ligament.)

**Fig 7:**
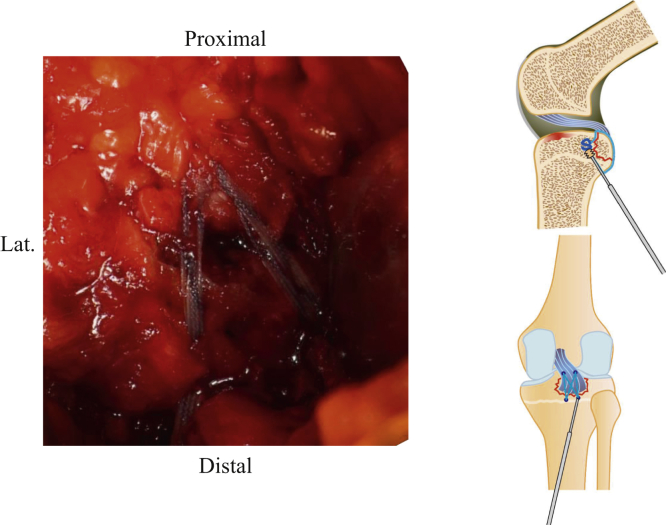
Reduction and fixing the avulsed fragment. The right knee is placed in a prone position on a standard operating table. The avulsed fragment is reduced by a 90° flexion of the knee joint. The sutures are then crossed and pulled distal to the periphery of the footprint and inserted with 2 anchors.

After incising the dorsal root of the oblique popliteal ligament and the joint capsule outside the dorsal root of the medial meniscus, the avulsed fragment and footprint can be identified (Fig 3). The footprint and the avulsed fragment are then dissected, and reduction viability is confirmed by threading the avulsed fragment and pulling it (Fig 4). Following this, 2 soft anchors (FiberTak Soft Anchor 1.8 mm; Arthrex Japan, Tokyo, Japan) are placed at the site (Fig 5). The suture is passed between the PCL, and the avulsed fragment is reduced by a 90° flexion of the knee joint (Fig 6). The sutures are crossed and pulled distal to the periphery of the footprint, which are then inserted with 2 anchors (SwiveLock 3.5 mm; Arthrex Japan) (Fig 7). The most important part of this technique is reduction of the bone fragment by 90° flexion of the knee joint. Pearls and pitfalls of this surgical technique are presented in Table 1, and the advantages and disadvantages of this technique are presented in Table 2.

**Table 1:** Pearls and Pitfalls of the Surgical Technique

Pearls	Pitfalls
By using the space between the medial head of the gastrocnemius muscle and the semimembranosus muscle, the procedure can be safely performed without processing the vessels.	To prevent the sutures from cutting the ligaments, multiple sutures are used, and they are crossed and fixed to the surface for better fixation.
Visualize the fracture fragment through a small incision and expand it widely as it is difficult to place the anchor perpendicular to the plane.	It is also difficult to simultaneously treat other injuries through the same incision in the prone position.
Because it can be operated under direct vision, control of the bone fragments is not compromised. This method is suitable for large or comminuted fractures.	
When repairing a bone fragment, the knee joint is flexed at 90° and the bone fragment is held down while pulling the thread tied to the fragment to ensure a secure repair.

**Table 2:** Advantages and Disadvantages of the Suture Bridge Technique

Advantages	Disadvantages
The anchor is driven into the bone, has a low profile, and does not require removal.	Large incisions are required
With the suture bridge method, it is possible to firmly fix the avulsed fragment to the surface and decrease the risk of destruction of the avulsed fragment.	Processing in the posterior compartment may jeopardize the posterior neurovascular structures.
The high-strength suture has a high mechanical strength, and the suture has a certain extent of flexibility.	Sutures may transect the ligament due to the thinness of the fixation sutures or too much tension on the sutures.

Postoperatively, our patient wore a knee brace for 4 weeks and was allowed only gentle range of motion training by a physiotherapist. Four weeks later, partial weight-bearing was allowed based on pain tolerance. The patient was allowed to return to sports in stages according to the recovery of muscle strength and stability of movement on the affected side.

## Discussion

Our study has 2 main key points. First, pediatric cases of PCL avulsion fractures are easily misdiagnosed on plane radiography; therefore, carefully physical examination and magnetic resonance imaging (MRI) should be considered. Second, using the suture bridge method, it is possible to reduce the risk of bone fragment destruction, fixing it firmly.

Swelling of the knee joint is often difficult to identify in PCL avulsion fractures in children, and the range of motion often is not restricted. Especially in the acute phase, posterior instability is often unclear. Although clinical findings such as posterior tibial sagging and provocative physical examination maneuvers such as the posterior drawer and quadriceps-active test are hallmarks of PCL insufficiency, no physical examination maneuvers can differentiate a substance tear from an avulsion injury. Therefore, radiographic analysis is critical for the diagnosis and treatment. Plain radiographs can be useful for detecting avulsed fragments if they are mostly osseous. However, osteochondral avulsions of the PCL can be missed on plain radiographs in skeletally immature patients. Therefore, the diagnosis was delayed in these 2 cases. If the patient who was hit in the anterior aspect of the lower leg showed positive posterior drawer sign and sagging, PCL avulsion fracture or PCL substance injury is suspected. MRI is useful not only for diagnosing fractures but also for assessing PCL substance and comorbid injuries.

The treatment options for PCL avulsion fracture depend on the degree of the injury. If the avulsed fragment is nondisplaced or minimally displaced, conservative treatment may be successful, with a focus on quadriceps strengthening. If the avulsed fragment is displaced or conservative treatment fails, surgical reduction and fixation may be considered. Toris^3^ reported 21 cases of delayed PCL avulsion fractures; 4 of them had residual anteroposterior instability. If the avulsed fragment is displaced, it is advantageous to attempt a reduction. This will reduce the risk of the child’s knees becoming loose and the prospect of poor function.

In previous reports, bone fragment fixation methods have been classified into screw-based^4^ and anchor-based.^5^ Disadvantages of the screw method include a risk of epiphyseal damage and destruction of the avulsed fragment. In contrast, the anchor method lacks the fixing strength. The suture bridge technique is commonly used for arthroscopic rotator cuff repair and is relatively easy to perform. It has been described as more effective for obtaining high initial fixation strength and increasing the contact area and contact pressure at the tendon footprint interface compared with the former techniques, such as the single-row or double-row technique.^6^ A study on the treatment of PCL avulsion fracture with high-strength suture fixation under arthroscopy has reported good results. The high-strength suture has a high mechanical strength, and the suture has a certain flexibility. The bone fragments are allowed to move slightly, which conforms to the principles of biofixation.^7^ Furthermore, the anchor is driven into the bone, has a low profile, and does not require removal. With the suture bridge method, it is possible to firmly fix the bone fragment to the surface and decrease the risk of destruction of the avulsed fragment. Operative management with open reduction and arthroscopic surgery often has been reported with similar good results in terms of complications and outcomes.^5^^,^^8^ However, in children, arthroscopic surgery was expected to be technically difficult due to the limited scope of operation.

In conclusion, pediatric cases of PCL avulsion fractures are easily misdiagnosed on plain radiography. However, careful physical examination and MRI should be considered if sagging or posterior drawer sign is positive after a strong hit to the anterior aspect of the lower leg. In children, ossification is incomplete, and they possess a lot of cartilage, so screw fixation easily destroys avulsed fragment. The suture bridge method can firmly fix the bone fragments. Furthermore, the risk of damage to the bone fragment is less, and a secondary surgery for implant removal is not needed.

## Supplementary Data

ICMJE author disclosure forms

Video 1 The patient is placed in the prone position on a standard operative table. A sterile tourniquet is then applied to the upper thigh of the leg to be operated. Surgery is performed using the modified Burks approach. A skin incision is made with the transverse limb just below the posterior knee crease smoothly curving into the vertical limb along the medial gastrocnemius. Deep fascia blunt dissection proceeds along the medial head of the gastrocnemius muscle, developing a plane between the semimembranosus tendon and the medial head of the gastrocnemius, with the latter retracted laterally to protect the popliteal vessels. The oblique popliteal ligament and posterior aspect of the joint capsule are incised lateral to the posterior root of the medial meniscus. After the footprint and bone fragment are dissected, 2 soft anchors (FiberTak Soft Anchor 1.8 mm; Arthrex Japan, Tokyo, Japan) are placed at the site. The sutures are then passed between the posterior cruciate ligament and the bone fragment, and the bone fragment is reduced in a 90° flexion of the knee joint. An anchor (SwiveLock 3.5 mm; Arthrex Japan) is inserted distally to the periphery of the footprint, and the sutures are pulled in to cross and firmly fixed on the surface. Postoperatively, the patient wore a knee brace. The patient was allowed to begin gentle range of motion activities and partial weight-bearing according to pain at 4 weeks postoperatively.
